# Inspiring respect for fathers as coparents through a trauma-informed, infant-family mental health transformation of community-based services: process and early implementation with a multi-agency community collaborative

**DOI:** 10.3389/fpsyg.2023.1282888

**Published:** 2023-12-13

**Authors:** James McHale, Donna Burton, Lisa Negrini, Alexandra Albizu Jacob, LaDonna Butler

**Affiliations:** Family Study Center, University of South Florida, Tampa, FL, United States

**Keywords:** coparenting, fathers, systems transformation, trauma-informed, family-centered, infant mental health

## Abstract

**Introduction:**

Despite compelling evidence that high-quality early care has an enduring impact, there has been little coordinated effort to transform services delivery to infuse Trauma-Informed Family Centered (TI-FC) principles into community-based agencies serving children and their families. A need for more culturally attuned, family-sensitive, evidence-based, and trauma-informed supports, especially for vulnerable children, their families and fathers, is apparent in evidence amassed by key stakeholders within the geographic area of this study. This report details the planning process, TI-FC training series, and organizational profile assessments. Authors conclude with recommendations regarding the establishment of multi-agency collectives, to include fathers, toward betterment of infant-family mental health at the community level.

**Methods:**

The current case study details the community-level transformational effort in which major health, mental health, substance abuse, and child welfare organizations serving families of children age 0-3 worked collaboratively to enhance TI-FC services. We describe a four-stage process (1 - planning, 2 - assessment of organizational readiness, 3 - surveys, document reviews and focus groups, 4 - delivery of a training series) detailing the work of the collaborative, guided by key agency decision-makers.

**Results:**

The study found significant initial success in adapting approaches to serving children 0-3 and their families through TI-FC perspectives. By proactively engaging several lead organizations in a deliberative planning process with universal aims and transformational principles, the collaborative team was able to coordinate organizational assessment, staff training and consultation, self-monitoring of organizational shifts, and problem-solving of obstacles and solutions to TI-FC services delivery.

**Discussion:**

All agencies succeeded in completing comprehensive, multi-faceted analyses of organizational culture, preparing personnel for TI-FC services through comprehensive training, and utilizing this collaborative to make deliberate and customized changes within their programs, as concerns both support of families and father engagement. Preliminary data indicate that important shifts took hold and signified changes across key domains of TI-FC care.

## 1 Introduction

According to the National Child Traumatic Stress Network (2016), trauma-informed child and family service systems are those:

…in which all parties involved recognize and respond to the impact of traumatic stress on those who have contact with the system, including children, caregivers, and service providers. Programs and agencies within such a system infuse and sustain trauma awareness, knowledge, and skills into their organizational cultures, practices, and policies. They act in collaboration with all those who are involved with the child, using the best available science to maximize physical and psychological safety, facilitate the recovery of the child and family, and support their ability to thrive (para. 1).

The dual focus on understanding trauma and celebrating and fostering strengths and resilience are each equally important. Families themselves, particularly families from nearly all marginalized, underserved ethnic and minority groups in the United States, are less likely to trust and engage in services provided by individuals and entities that view them as vulnerable, broken, or “less than” (Bocknek et al., 2017). Strategically helping service agencies and providers to recognize the importance of strengths-based approaches–at the same time as they are upskilled to recognize and respond more sensitively to historical and present-day trauma, adversity, and stress impacting infants, young children, fathers, and families–is an exigent and formidable task. However, with a collaboration of key community partners and a collective impact lens, transformational attainments in family service systems are an achievable goal.

Cultivating a family-strengths orientation that proceeds from a trauma-informed frame is not instinctive and is best seen as a work in progress. In customary practice, agency personnel have been trained to see and record evidence of men’s absenteeism and violence potential. Such perspective and due documentation create a bias to view fathers, at best, as weak and requiring help to remedy their failings – and, at worst, as neglectful or as purposeful perpetrators of trauma and harm toward their young children. Indeed, even the very act of guiding agencies and agency personnel to systematically screen for trauma can heighten bias for singling out problems and their aftereffects. Further, most men do not respond well to inquiries about susceptibility to trauma and suggestions of vulnerability (McHale and Jenkins, 2023). And, assumption of a pathology lens can be problematic and even disruptive if agencies do not possess the proper resources to afford responsive follow-up once historical or ongoing trauma has been uncovered. Tight-knit resource and referral pathways in service systems can alleviate some of the burdens felt by individual agencies and organizational entities, but only as far as an organizational culture has evolved to provide adequate supervisory and accessible backup support for front-line personnel in their direct everyday dealings with fathers and families. Internal policies and procedures for such backup and self-sustaining mechanisms enabling upper and middle management to reflect, monitor, and replenish can all be crucial determinants of the sustainability of trauma-informed, family-centered practices and transformations within systems.

Reflecting on the NCTSN principle that child- and family-serving agencies must act with all involved with the child, constraints and limitations within agencies and service systems abound. As has been well-chronicled throughout the professional literature, most infant- and young-child-serving agencies have historically positioned themselves to initiate and maintain contact with one and only one informant within the family when an infant or young child is identified for services (McHale and Phares, 2015). Almost invariably, points of contact for children birth to age 3 are children’s mothers, though certainly identified clients can be fathers, grandparents, foster parents, or other caregivers. However, the NCTSN tenet that agencies engage all involved with the child is rarely achieved.

The reasons are legion. Organizational policies, documentation and billing procedures and constraints, harmful stereotypes characterizing lower income and nonresidential fathers principally in terms of their failings, the conspicuous absence of professional competencies among agency staff for comfortably and knowledgeably engaging and working with fathers and mothers as coparents – simultaneously - and other unnoticeable constraints in organizational, funding and service system structures combine to militate against instituting a true family-centered approach (Lu et al., 2010). In response, infant-family mental health perspectives and best practice approaches have begun calling for an assessment of and attention to the child’s full coparenting and caregiving context in offering effective client (infant, family) centered services (Zeanah and Lieberman, 2016; McHale et al., 2023).

It was within this zeitgeist that a transformative cross-sector community initiative spearheaded by a university-based Family Study Center (FSC) in the Southeast United States, hereafter referred to as the Trauma-Informed Family-Centered (TI-FC) Collaborative^1^ was established. The Collaborative set out to reimagine the overall scope and delivery of services to families by bringing TI-FC care and practices to the center of the region’s infant and early childhood services landscape. A dawning collective awareness throughout the county and the state had begun acknowledging the unparalleled importance and impact of children’s earliest years, a recognition reflected through numerous state and local efforts and initiatives designed to support the foundations of early learning.

The FSC envisioned a collaborative through which transformative efforts would result in a platform from which to launch the new directions for scope and delivery of services to children age 0–3 and their families. The inroads to inter-agency collaboration began by gathering partners to collaboratively consider and agree upon terminology and ideas. The FSC’s history as a convenor for community partnership initiatives concerning the unparalleled importance of the first 3 years of every child’s life offered a common starting place for consensus on critical descriptions. It its first meeting the TI-FC Collaborative agreed upon key terms, concepts, definitions and operationalization. Cross-partner dialog resulted in the following:

- By trauma, we mean deeply distressing or disturbing experiences that usually include an emotional response to a terrible event such as abuse, violence, (including domestic violence, sexual violence, etc.), accidents or a natural disaster. Trauma can be defined as a single event, series of related or unrelated occurrences, or chronic and overwhelming stressors within an environment.
- By trauma-informed (TI) care, we mean services that consider the impact of trauma and the often-complicated paths to healing and recovery. Trauma-informed care includes specific policies and practices that identify, incorporate, and remain sensitive to an individual and/or family’s trauma history, symptoms, strengths, and coping with overwhelming emotion. The goal of TI care is to avoid re-traumatizing the individual while creating an environment of safety, healing, and empowerment that ultimately helps individuals and families make meaning of their trauma. Trauma-informed care requires changes at every level of the organization to achieve full implementation.
- By infant mental health we mean infants’ and very young children’s ability to experience emotions, develop relationships and learn. Key to preventing and treating mental health problems of very young children and their families is an approach informed by infant mental health principles and practices, with supports for relational health enabling development of healthy social and emotional behaviors. Infant-family mental health is best promoted by intentional and successful strengthening of the relationships among the important caregiving adults (“coparents”) responsible for the child’s care, upbringing, and social–emotional development.
- Finally, with human services agencies increasingly supplementing and supplanting deficit-based practices (prioritizing problems and needs) with strengths-based approaches in work with children and families, this project operationalized strengths-based approaches as valuing strengths, skills, connections, potential, and capacity for growth, with each organization reflecting internally on applications of these principles in their own change efforts.

**Figure 1:**
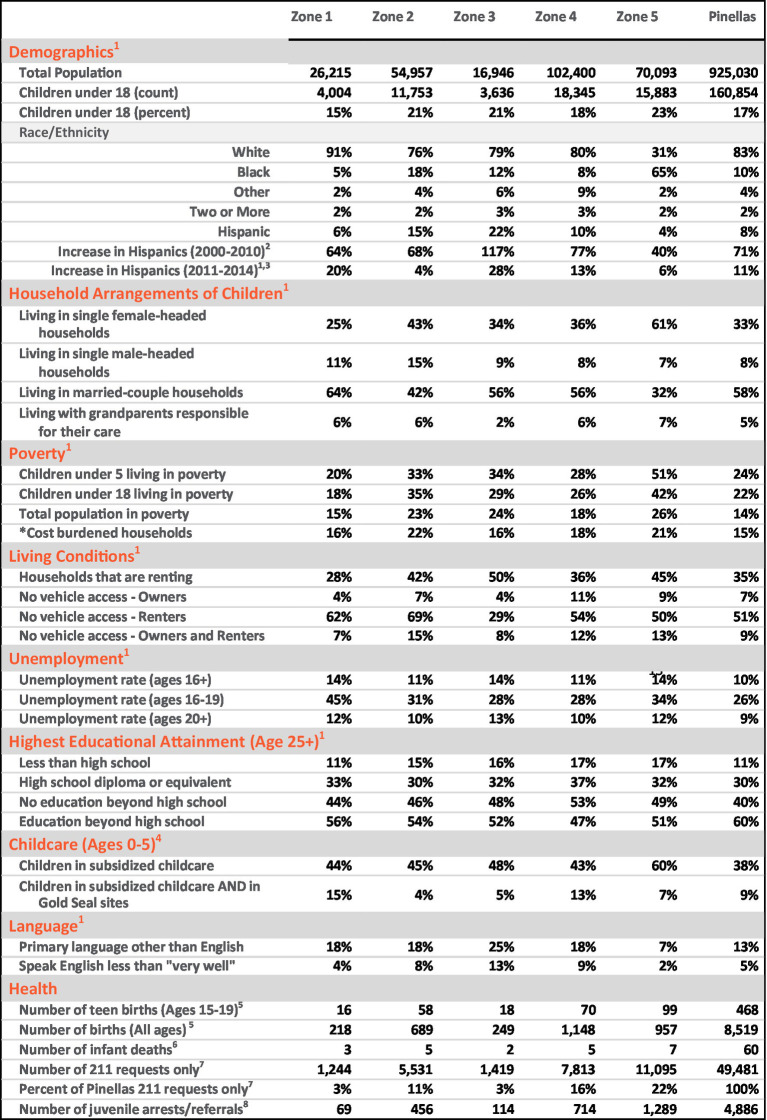
Differential Risk in Zone 5. (south St. Petersburg, Pinellas County). *Household income less than $20,000 AND spending more than 30% of income on housing. Sources: ^1^American Community Survey 5-year Estimates ^5^FloridaCHARTS Birth Query System ^2^Decennial Census ^6^FloridaCHARTS Infant Mortality Query System ^3^American Community Survey 5-year Estimates ^7^2–1-1 Counts Tampa Bay Requests (not total calls) ^4^Early Learning Coalition JWB Data Uploads ^8^Florida Department of Juvenile Justice.

Despite agencies’ concurrence that high-quality early care can have enduring impact (Haskins, 1989; Heckman, 2011), no coordinated effort to transform systems of care to infuse TI-FC principles into standard multi-agency ways of work had previously been undertaken. A need for more culturally attuned, father- and family-sensitive, evidence-informed trauma-informed supports and services - especially for the area’s most vulnerable young children and families - had become starkly apparent in countywide geographic data (Warren, 2013; Figure 1).

As evident from Figure 1, risk determinants are not proportionally distributed throughout Pinellas County. Rather, in a manner paralleled in communities throughout the United States, significant sectors of young children and families disproportionately experience substantial and substantive risk, inferior quality of care, and unmet health needs. Not surprisingly, concurrent disparities are also documented in early socioemotional and early physical health outcomes (Pinellas County Access to Health Profile, 2015). These data are wholly consistent with the growing recognition of the relationship between neighborhoods and health, where zip code has been recognized as a stronger predictor of a person’s health than their genetic code (Graham et al., 2015).

When the current initiative began, amassing scientific data and targeted communications had begun illuminating how early, inadequately addressed stress and adversity weigh heavily and inordinately on young children and their families, adversely impacting children’s thriving and readiness to learn by kindergarten age (Zeanah, 2009; Shonkoff, 2010). From an agency service perspective, in circumstances where young children’s emotional health is jeopardized, intensive family support is called for to help the child move back onto a positive developmental trajectory. High quality, accessible and culturally attuned services are indispensable in communities that contend with a high concentration of environmental stress owing to poverty, racism, disenfranchisement, and trauma. Such were the circumstances challenging many families with young children in the Florida community that is the focus of this report (Warren, 2013).

Buoyed by this converging evidence, the TI-FC Collaborative assumed a TI-FC lens for service provision as its collective aim. The initiative was conceived and coordinated through the joint efforts of a small collaborative of leading service providers in the community for greater initial impact. Goals were to transform how major child- and family-serving agencies approached their work with fathers and families so that there would be no “wrong door” – that is, any family with a young child who received supports and services from a program or an agency established to serve them could expect to be met with a culturally competent and humble, respectful and authentic set of supports that (a) recognized and validated the family’s love and ongoing efforts to support the child (b) recognized and knew how to sensitively address challenges to father (and mother) engagement, and seeming resistance to treatment that had its roots in trauma histories (c) saw and supported the child within the context of their full family support network, instead of directing all services and supports to and through the child’s mother or primary caregiver alone and (d) recognized when family needs outpaced the existing capacities of the provider or agency to call upon known, connected community partners to help adequately redress unmet needs.

Though no community transformation blueprints existed, the FSC and partner organizations representing maternal and child health, home visiting, substance abuse, and child welfare set out to create a coordinated, systematic, and comprehensive framework drawing on the evidence-based practice of early childhood mental health consultation (ECMHC; Perry et al., 2010). The FSC assembled an expert team to support agencies in reviewing their policies, procedures and networks and adjusting their already effective and evidence-based intervention models to systematically incorporate TI-FC practices into the routine care and services afforded to families. Partners all agreed to begin the work evaluating their organizational readiness for implementation of new services and/or best practices in TI-FC care, to use these baseline data to guide transformational efforts within individual entities and to strengthen referral channels among them.

The structure, process and early implementation of these efforts are outlined in the sections that follow, with particular attention given to details of the procedures of the planning sessions to illuminate key elements of the Collaborative’s planning.

## 2 Method

### 2.1 Participants and setting

The community collaborative was organized by a university-based, community-engaged Family Study Center, whose mission includes the development of family-sensitive models that help promote infant-family mental health regionally, statewide, and nationally. Collaborative partners whose local efforts on behalf of families were essential in the transformation that this initiative sought to achieve included home visiting programs -- specifically those in maternal and child health; pediatric medical homes, including Community Health Centers; child welfare initiatives, specifically the foster/relative caregiver care system; and mental health programs and agencies. While designed as an inclusive multi-sector effort that could increasingly incorporate multiple additional providers, agencies, and organizations in training and support efforts core to the transformative work, five organizations took leadership as core strategic partners in the initial planning and implementation of the transformative initiative:

- Pinellas County Health Department, Maternal and Child Health Division- PCHD’s Maternal and Child Health Division (MCHD)- Healthy Families, Nurse-Family Partnership, WIC, and Healthy Start
- Community Health Centers of Pinellas at Johnnie Ruth Clarke- Family and Pediatric Medicine, Obstetrics and Gynecology Care.
- Adoption Related Services- Mental Health Organization serving biological, foster, and adoptive families in Pinellas County
- Operation PAR- Pinellas County’s lead Substance Abuse Treatment organization
- Healthy Start Coalition-the county’s Maternal and Infant Home Visiting network

Dedicated funds were provided by the sponsor of the Planning Grant to cover the administrative costs of regular attendance by agency leaders and decision-makers at planning meetings. The participation of leadership was viewed as essential in supporting staff motivation and “buy-in” of the TI-FC changes that would be advocated and encouraged internally. Funds also supported special data collection and collation efforts carried out by partner agencies to provide the process and output indicators enabling assessment of project impact.

Agencies organized around the objective of helping their organizations develop trauma-informed, family-centered practices. The collective aim was to create a service system that better promoted families’ capacities to furnish stable environments and strong, secure relationships that would support the growth and thriving of the county’s young children. An interlocking aim was to assure access to resources that would help with challenges they faced and would face. Core strategic partners agreed to participate in a common set of development activities, brokering Memoranda of Understanding to collaboratively transform ways of working with families with young children in the community. Specifically, agency partners agreed to:

- Participate in monthly leadership meetings involving all partner organizations.
- Undertake an organizational self-assessment to determine readiness for a trauma-informed, infant-family mental health initiative.
- View the self-assessment as an iterative living document to undergo intentional edits, informed by the organization’s participation in the initiative, at two follow-up timepoints
- Establish a universal family-level trauma screening for all programs serving children birth to 5 years.
- Mandate that program staff participate in trauma-informed practice and coparenting/ family-centered practice training that focused on effective engagement and work with men and fathers.
- Mandate that all supervisors and managers take part in Reflective Supervision training, toward the goal of enhancing the agency’s commitment to reflective practice.
- Create policies and procedures regarding agency use of trauma-informed practices, family-centered services, and infant-family mental health approaches.
- Work with the initiative to establish a streamlined referral and linkage system for families with children birth to 5 years needing infant-family mental health clinical services.

### 2.2 Procedures

The study design was rooted in an *interactive approach* to *formative evaluation* to ensure that the collaboration activities among community partners were feasible and appropriate when held up to the stated objectives for frameworks for change within partner organizations. Whether partner agencies were developing new activities or adapting and modifying existing services, the formative evaluation was designed to improve models for change over time. Moreover, with the knowledge that efficient change within organizations happens through a combination of top-down and bottom-up approaches, administrative leads and agency staff were engaged in a range of data collection methods, providing opportunities for equitable sharing of information on critical organizational operations, as well as change efforts over time. Activities within each of the four stages of the process proceeded as follows:

*Stage 1*: Funding was obtained to compensate key agency decision-makers to take part in a 5-month planning stage. During the planning phase, agency leaders met bi-weekly to define terms and objectives, outline the scope of the initiative, agree on a common design, and set of commitments, plan communications with agency personnel and concretize a strategy for regularly reviewing progress. Meetings were facilitated by the first and third authors; the third author worked regularly and collaboratively with a university-based program assistant to serve as the dedicated administrative liaison for the project coordinating team.

*Stage 2*: This stage consisted of an Organizational Readiness Self-Assessment in which collaborative partners invited agency or unit staff to participate in or contribute to four data collection activities addressing TI-FC care within their organizations. Timelines for the major OSA activities are summarized in Table 1, and an overview of the Method including evaluation details and participant survey numbers is provided in Table 2. A forthcoming manuscript considers sampling, data quality and representativeness, and nonresponse bias in greater detail. Specifics of the various activities completed are outlined below:

**Table 1:** Timeline for TI-FC stages 2 (organizational readiness self-assessment) and 3 (OSA profile development and dissemination) activities.

Activity	Year one				Year two			
	OCT-DEC 2018	JAN-MAR 2019	APR-JUN 2019	JUL-SEP 2019	OCT-DEC 2019	JAN-MAR 2020	APR-JUN 2020	JUL-SEP 2020
Organizational readiness self-assessments								
OSA–staff survey	X				X			X
OSA–administrative survey	X				X			X
OSA–document review		X	X	X	X	X	X	X
Creation of organizational profiles from OSAs								
OSA–development and updating of partner organization profiles			X			X		X
Agency staff focus groups								
Conduct focus groups regarding perspectives on TI-FC care at agency					X	X		
Analyze focus group results and disseminate recommendations						X	X	
Final reports to organizations on all evaluation activities								

**Table 2:** Summary of evaluation activities.

Methods	Relevant data
Surveys. Surveys were conducted at 3 time points, online through Qualtrics. Response choices were presented as 5-point Likert Scales, with response options ranging from Completely Agree to Completely Disagree Staff survey participants: Counselors/therapists Case managers/home visitors Administrative support staff Medical staff Other Administrative supplemental survey participants: Supervisors Directors/Executive management	Staff Survey Baseline: (*n* = 204) Midpoint: (*n* = 210) Project End: (*n* = 196) Administrator Supplemental Survey Baseline: (*n* = 47) Midpoint: (*n* = 56) Project End: (*n* = 34)
Document reviews. Partner agencies provided access at 3 time points to documents reflected organizational efforts regarding: service planning and trauma-specific services; trauma screening and assessment; treatment, referral, and discharge planning; client engagement and representation; administrative support and training; and program evaluation.	Documents reviewed from partner organizations (*n* = 5) at three time points
Focus groups. Focus groups were conducted to supplement organizational profile data on key elements of progress toward TI-FC capacity. Participants were volunteers from agency direct-service staff. A report summarizing focus group topics, themes, summary, and recommendations was presented to the collaborating partners at project end.	Focus groups Agencies: (*n* = 4) Total participants: (*n* = 38)
Evaluation methods culminated in the development of *Organizational Self-Assessment Profiles* These profiles were iterative individual agency profiles documenting key indicators of readiness to implement, modify, or enhance trauma-informed family-centered care. Profiles were presented to agency leads at three times points and reviewed in meetings with the evaluation team. Progress in developing TI-FC agencies was documented and quantified along key TI-FC domains. Separate sections were dedicated to strengths, areas for improvement, and a plan of action for each partner organization.	

First, a *program staff survey* was disseminated to eligible program staff; eligible staff were personnel within partner organizations that had direct contact with and knowledge of the clients served within the 0–3 programs and services. The staff survey was adapted, with permission from the authors, from a Trauma-Informed Care Organizational Survey, developed at the University of South Florida (Hodges et al., 2014). Survey participants were identified by agency partners, and specifically those key leaders attending the meetings of the TI-FC Collaborative. These agency leads were seen as change agents with knowledge of two critical pieces of information: (1) the mission and objectives of the Collaborative; and (2) persons within their agencies well-suited to providing information about the agency and its clients. They were therefore asked to disseminate survey links by email to agency staff and to provide encouragement and prompts to complete the survey. The staff survey addressed seven domains of TIC, including:

Domain 1: Competent Trauma-Informed (TI) Organizational and Clinical Practices

- Sample item 1: My agency offers trauma-specific, evidence-based practices.
- Sample item 2: Staff members use a strengths-based, person-centered approach in their interactions with clients and their families.

Domain 2: Client and Family Engagement in TI Care

- Sample item 1: Clients and their families are routinely involved in treatment and/or service planning.
- Sample item 2: There are systematic opportunities (beyond satisfaction surveys) for clients and families to give feedback regarding TI care.

Domain 3: Father and Coparent Engagement in TI Care

- Sample item 1: My agency prioritizes active outreach to fathers and coparents and includes them in case planning and services provided.
- Sample item 2: I receive the encouragement, support, guidance, and training I need from my agency for working with fathers and coparents with TI care needs.

Domain 4: Organizational Readiness for TI Care

- Sample item 1: Leadership in my agency ensures that all staff are prepared to offer TI care in culturally responsive and appropriate ways.
- Sample item 2: My agency provides the resources (technology, staffing, and training) for implementation of TI care.

Domain 5: Vision for Services

- Sample item 1: Trauma-informed care should be offered within all the agencies programs and services.
- Sample item 2: All staff should be informed on TI care and knowledgeable about delivering TI services.

Domain 6: Training, Knowledge, and Skills

- Sample 1: I have received the training I need to participate in delivering TI care.
- Sample item 2: My background, education, and experience are a good match for providing TI care.

Domain 7: Trauma-Informed Care in the Community

- Sample item 1: Trauma-informed evidence-based practices are accessible to children and families in my community.
- Sample item 2: My community is committed to developing a trauma-informed workforce.

The *program staff survey* was conducted at each partner organization site three times: at baseline, 12 months (midpoint), and project end. Concurrently, a *supplemental administrative survey*, was completed by partner organization administrators at the same three time points. Participants completed the surveys via Qualtrics. The supplemental administrative survey, which was adapted from the *Creating Cultures of Trauma-Informed Care CCTIC Fidelity Scale* (Fallot and Harris, 2015), directed program administrators to describe program indicators reflecting five core values associated with a culture of trauma-informed care (safety, trustworthiness, choice, collaboration, and empowerment; Harris and Fallot, 2001a,b). The CCTIC includes six domains, each incorporating subdomains corresponding to the five core values. The six major CCTIC domains are:

1. Program Procedures and Settings;
2. Formal Services Policies;
3. Trauma Screening, Assessment, and Service Planning; Trauma-Specific Services;
4. Administrative Support for Program-Wide Trauma-Informed Services;
5. Staff Trauma Training and Education; and.
6. Human Resources Practices.

The objective of all surveys was to seek information from program staff and administrators about their experiences in the identified areas of TI-FC care to determine whether their experiences were consistent with the proposed model for systems transformation. All surveys were disseminated via emails sent by the university-based administrative liaisons for the project coordinating team to the community partner administrative leads. They were considered census surveys with no exclusionary criteria. Participation was voluntary and anonymous, and no incentives were offered for participation. No major risks were projected, and no adverse events were reported. Procedures were reviewed by the USF IRB, and exemption from signed consent was granted. Each survey took approximately 15 min to complete.

Microsoft Excel was used to organize data obtained through Qualtrics. The survey data were analyzed using SPSS 25. Descriptive statistics (e.g., mean scores, item response frequencies) were obtained along with the characteristics of participants. Surveys from participants who did not complete questions beyond the second domain were treated as incomplete and excluded from group analyzes.

Also in Stage 2, a *document review* was conducted. Documentation is valuable as a method of program evaluation because it provides an historical context for change, relies on readily available and unbiased information, and does not interrupt staff routines related to client care. The documents reviewed pertained to key program components associated with a culture of trauma-informed care, including trauma screening, assessment, service planning, and trauma-specific services; administrative support, involvement of persons served/peer representatives; staff training, education, and support; and program evaluation (Fallot and Harris, 2015). Participating agencies provided the second and fourth authors with common public documents describing the nature of their programs and service activities (particularly related to TI-FC care) for review. The materials submitted provided documentary evidence of the presence or absence of policies, procedures, and organizational functions of material interest to the process evaluation component of the study (e.g., policies and procedures related to trauma-informed intake and assessment; staff training and development).

Finally, a smaller subset of staff from each organization participated in *focus groups* designed to obtain direct and first-hand confirmatory information about staff experiences with the implementation of TI-FC strategies and to determine whether services, as portrayed in census surveys, were on-target and amenable to planned enhancements - which included implementation of a universal family level trauma screening for adults and children birth-3 years. The study team developed and implemented the schedule of focus groups with agency staff as participants.

Focus groups are useful in exploring topics in depth and in this case providing essential perspectives from people served. Focus groups offer a quick, reliable way to gather information on common impressions and ensure range and depth of information (Barbour, 2007; Liamputtong, 2011). Program staff were ideally situated to provide insights into TI-FC care over time. The broad topic areas discussed included participants’ experiences in various programs and the delivery of services, their impressions of TI-FC care within their respective programs, and the degree to which their experiences matched the program as it was intended. For example, they were asked to describe services, policies, and protocols to assess if said services operated from trauma-informed perspectives. Initially, it was anticipated that focus groups would be conducted at two time points, with the intent of learning how the activities of the TI-FC Collaborative impacted services and the experiences of program staff over the duration of project efforts. However, the second focus group was not completed due to the COVID-19 pandemic and related obstacles.

Focus group participants were identified and recruited by partner agency administrative leads (consistent with the requirements for the protection of privacy), with the goal of recruiting a minimum of 6 to 8 participants per focus group. Verbal consent was requested and given at the start of each group, and discussions were audio-recorded with the permission of the participants. Each focus group lasted 60–90 min (about 1 and a half hours). Focus groups were conducted at partner agencies’ offices for participants’ convenience. In total, 30 staff participated in the focus groups across four agencies. A wide range of perspectives were represented across the groups as staff roles ranged from direct care staff and program supervisors to executive leadership. Participating staff indicated varying lengths of employment at their respective agency, with employment periods ranging from less than 1 year to 28 years.

Two evaluation staff facilitated the focus groups, with one member as the primary moderator and the second as a facilitator/recorder. The evaluation team then analyzed findings and disseminated results to collaborative partners. Honoring the time-compressed nature of the evaluation timeline, the team completed rapid thematic analysis of the data so that findings could efficiently inform practice, i.e., content development for staff trainings. Rapid thematic analysis is an evidence-based qualitative approach commonly used in health pragmatics research (Renfro et al., 2022). Results from focus groups helped to augment organizational profiles for key aspects of TI-FC capacity.

*Stage 3*: In stage 3, data from the completed *surveys*, *document reviews* and *focus groups* were collated to inform development of partner organizational profiles, which completed the Organizational Self-Assessment (OSA). These profiles were developed by each partner organization in collaboration with the study team and the first served as a baseline against which the organization could later assess transformational shifts. OSAs resulted in individual agency profiles of key indicators of readiness to implement, modify, or enhance TI-FC care. The baseline OSAs were first updated at 12 months, and then again at project end, for each agency partner. In this way, the OSA was able to illustrate for programs how they were improving over time, brought to light areas for continued quality improvement, and helped to engender plans for continued growth and development going forward.

*Stage 4*: In the fourth stage, which launched soon after the initial OSAs were completed and shared with agency leaders, all front-line staff, managers, and supervisors were required by their organizations and programs to participate in a coordinated series of TI-FC trainings for multi-agency staff. Trainings addressed universal trauma-informed practices, infant-family mental health, father engagement and coparenting and reflective supervision and practice. The university-based administrative liaisons scheduled the trainings for the project coordinating team in collaboration with the lead representatives from each partnering agency. Forethought was given to rotating the sites for the trainings held in the community at the various participating agencies. Multiple offerings of each training module were arranged, and each training session was made accessible to members from all partnering organizations to maximize flexibility.

The unusual composition of the multi-partner collaborative elevated the memorability of these trainings, as – following presentation of relevant core content by the university-based content experts (the first and fifth authors) - staff from different agencies would reflect as a group on the current state of practice within their spheres of operation. Staff would describe typical practice, experiences of better or best practice, and experiences of falling short of the mark. Together partners would reflect on one or more areas for immediate adjustments within their own organizations and services and present these publicly to the other organizations in attendance so multiple organizations could hear the analyzes of changes, big and small, that others anticipated being able to make. As the last exercise, partners were instructed to project forward to simple but larger procedural adjustments that might make practice changes more enduring and sustainable.

The thrust of the initiative was that mindfulness about obstacles – agency-wide, family-specific concerns and challenges and personal (blind spots and biases) - were all part of the formula and solution for transformational change. For this reason, the presence of upper and middle management supervisory staff as participants together with front-line providers at the large-group training sessions and convenings helped build solidarity. It also provided multiple perspectives from which others learned. Training content was later enshrined in a series of short recordings and manuals made accessible to project partners for use in future onboarding and training of new staff in strengths-based approaches. Throughout the initiative, the university-based study team took responsibility for planning and directing all activities. In planning, they creatively combined qualitative and quantitative methods of both process and outcomes activities to best meet the needs of short-, mid-, and long-term goals for organization-level as well as systems change. The evaluation results were reported to project leads and community partners in a timely manner. In fact, lead evaluators attended the monthly leadership meetings to actively observe the transformational process in real time, and to provide reports of progress of and findings from evaluation activities.

Formative information and outcomes guided the consultation and feedback provided to partner organizations on elements critical to the development of TI-FC care and related programs and services. Addressed were training plans (including protocols for onboarding new staff), policies and procedures, intake and screening processes and materials, interventions offered, and referrals made. This intensively collaborative process enabled organizational leads and agency partners to make use of their own evaluation results to best determine opportunities for procedural shifts in TI-FC care. Study activities were hence deliberate and intentional in assisting each partner organization to consider how they might strengthen approaches, add interventions where needed, and improve outreach to and engagement of all caregiving adults coparenting children prenatal to age 3, specifically men and fathers but also other engaged family caregivers.

## 3 Results

This section summarizes findings from the organizational self-assessments (OSAs) and from additional corroborating data collected from the participant organizations on the state of TI-FC care at their agencies at various stages of the initiative. We first present select findings from OSA staff surveys at various junctures of the initiative capturing the overall momentum of cross-time change. This is followed by illustrative results from the OSA administrative supplemental surveys and the OSA focus group sessions. Rather than providing agency-by-agency findings to best reflect system transformation, we summarize the trends seen upon combining data across all partner agencies. Overall, both survey findings and additional qualitative data collected over the course of the initiative (which were also broken out into separate reports for each participating agency) reflected commitments from staff across agencies to streamline referrals and services and transform their system to become trauma informed.

OSA Survey – As described in Table 2, the OSA Survey included a Staff Survey and an Administrative Supplemental Survey. Results of the two surveys are detailed separately below.

### 3.1 Findings from OSA staff surveys

**Figure 2:**
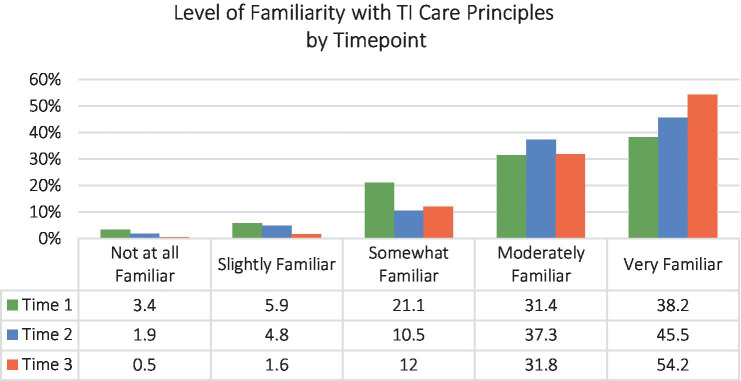
Familiarity with trauma-informed care principles by timepoint.

Our chief interest in examining survey data from staff was establishing if there was an increase in uptake of TI-FC principles across the initiative’s duration. Figure 2 depicts familiarity of agency staff, collated across all participating partners, with TI care principles at the beginning, middle and end of the initiative. Survey data showed that staff levels of familiarity with targeted principles improved over the project’s life.

**Figure 3:**
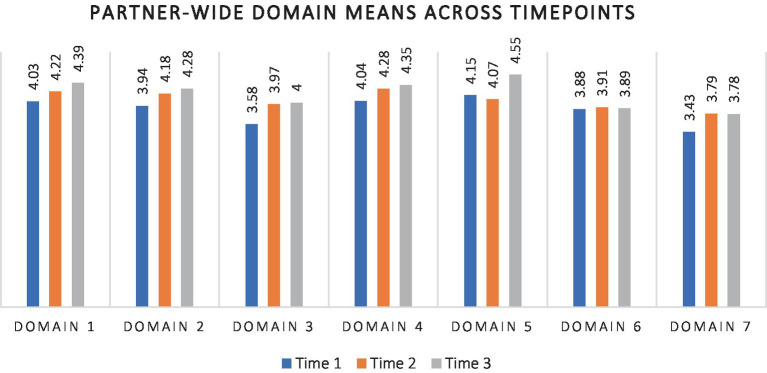
Partner-wide domain means across timepoints.

Next, we examined what agency staff had to say about their familiarity with the content of each of the different domains targeted in the OSA surveys. Figure 3 summarizes levels of understanding reported by agency staff across the 7 key domains (Competent Trauma-Informed (TI) Organizational and Clinical Practices; Client and Family Engagement in TI Care; Father and Coparent Engagement in TI Care; Organizational Readiness for TI Care; Vision for Services; Training, Knowledge, and Skills; and Trauma-Informed Care in the Community).

As can be seen from Figure 3, comparison of mean scores over time for the 7 domains suggests gradually higher scores for Domains 1 through 5 at Times 2 and 3. There was also a higher mean at Time 2 than at Time 1 for Domain 7, but no further elevation at Time 3. Only on Domain 6 (Training, Knowledge, and Skills) did mean scores appear unchanging across time, perhaps because newly onboarded staff who had not partaken of the training series were among those completing surveys at later time points. It is also possible that the seeming lack of organizational change from Time 2 to Time 3 for Domains 6 and 7 may have been because they were the last two domains presented in the survey. More respondents submitted only partially complete surveys at the time of the third administration. Since analyzes were run Domain by Domain, the effective sample size would have been smaller for incomplete Domains, potentially affecting the overall result pattern.

Once available, summary data were presented to and discussed with agency partners in group consultation. Afterward, each agency was provided with organization-specific results capturing the shifts depicted just for their own entity. This allowed each organization to reflect upon and make decisions for future internal action and change, based on their own profile. Overall, based on staff survey results, the TI-FC Collaborative partners as a group were encouraged to consider the following:

- Continuing efforts to revise and develop policies and augment staff capacity-building efforts (e.g., training, reflective supervision) beyond the TI-FC initiative.
- Reviewing specific survey domains and their indicators to fine-tune organization-level policies and protocols related to TI-FC care
- Addressing strengths as well as areas of improvement that emerged from the survey to help advance the mission of systems transformation in TI-FC care
- Continuing with training plans for each unit and staff member such that each person’s training, knowledge, and skills in the targeted domains continued to grow;
- Ensuring that onboarding of all new staff included comprehensive training in TI-FC principles (as noted, presence of fresh staff at Times 2 and 3 may have contributed to the relative lack of cross-time change seen in Domain 6)
- Continuing efforts to engage the community in TI-FC principles (as apropos to the relative lack of change seen in Domain 7)

### 3.2 Findings from OSA administrative surveys

The *Organizational Self-Assessment Administrator Supplemental Survey* highlighted program administrators’ experiences in six identified areas of TIC (Program Procedures and Settings; Formal Services Policies; Trauma Screening, Assessment, and Service Planning and Trauma-Specific Services; Administrative Support for Program-Wide Trauma-Informed Services; Staff Trauma Training and Education; and Human Resources Practices) to document whether their experiences in those realms were consistent with the TI-FC Collaborative’s proposed model for systems transformation. The survey augmented previously reported data from the OSA Staff Survey (above) and yielded additional insight into the TI-FC Collaborative’s efforts to become a more trauma-informed provider network. The following key findings from the Administrative Supplemental Survey highlighted advances toward becoming more trauma-informed over the initiative.

**Figure 4:**
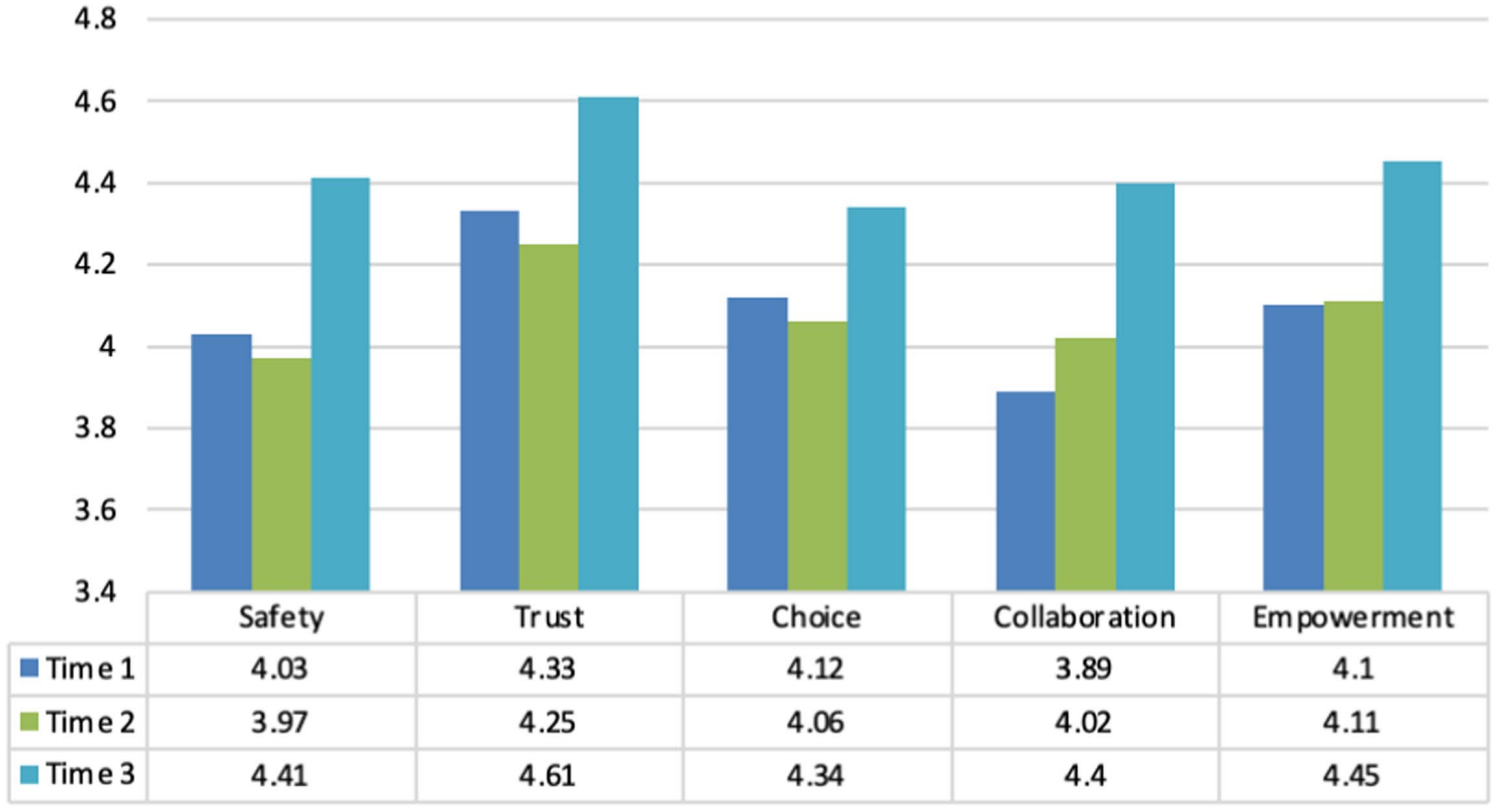
Domain 1 (program procedures and settings) sub-domain mean scores.

- Comparison of partner-wide level of familiarity with TI principles overall showed a cross-time decline in those reporting being only slightly familiar or not at all familiar.
- Commensurately, the proportion of partner-wide administrative respondents who reported being *moderately to very familiar* with TIC principles advanced steadily over the course of the initiative, climbing across the three time points - 72% at Time 1, 89% at Time 2, and 97% at Time 3.
- A comparison of administrator reports on the CCTIC over the course of the TI-FC initiative (Figure 4) indicated that on Domain 1 (Program Procedures and Settings), mean subdomain scores for the five core values of TI care (i.e., safety, trustworthiness, choice, collaboration, and empowerment) changed only modestly from Time 1 to Time 2. Still, all showed notable mean level changes by Time 3.

The progression noted in Figure 4 is considered particularly auspicious. In that pronounced initiative effort was invested in addressing transformations of program procedures and settings, advancements in administrators’ mean scores for all 5 subdomains was important. Because those in administrative or leadership positions often have more longevity in their agencies and possess greater familiarity with TI-FC care principles, they are well-positioned to foster an infrastructure and environment needed to strengthen their organization’s internal efforts toward becoming more trauma-informed and family-centered.

As with the OSA staff results, administrator results were also presented to and discussed with agency partners so each agency could reflect on both trends across the system or care and on their own organization-specific results, enabling informed decisions for future internal action and change based on their own entity’s profile. Overall, based on administrative survey results, TI-FC Collaborative partners were encouraged as a group to:

- Review specific survey domains and their indicators to fine-tune organization-level policies and protocols related to TI-FC care and ensure that agency leadership took a leading role in moving the agency forward regarding these practices.
- Address the strengths as well as areas of improvement that emerged to help advance the mission of systems transformation in TI-FC care. Partners were directed to look most closely at the highest and lowest endorsed items. This helped them to identify specific training needs and to develop strategies helping assure that key domains and subdomains became an integral component of agency-wide meetings (formal and informal).
- Work to streamline the continuum of care across partners and, therefore, improve services available to the community.

### 3.3 Findings from focus groups

Whereas participant knowledge and awareness of the initiative’s purpose and goals varied within and across agencies, most demonstrated at least a perfunctory understanding. Most frequently referenced in responses was the helpful nature of the trainings. This undoubtedly owed to the training series as the most overt exposure staff had with the initiative. Specific topics/themes that emerged from focus groups included:

#### 3.3.1 Training, skills, knowledge

Participants highlighted numerous benefits to the trainings, especially how they increased staff awareness of the widespread impact of trauma. Staff valued how trainings provided them with tools and language to inform how they approach and discuss trauma with clients. They also commented on how their own trauma could influence their well-being and ability to serve clients. Most pertinent to the focus of this special issue, they spoke to their new insights regarding the importance to children of actively involving fathers and other coparenting family caregivers in services.

#### 3.3.2 Observed changes

Participants across all groups said that they saw benefits and changes occurring within their agencies due to the initiative. Several observed modifications in the continuum of care such as changing screening procedures and forms to be more trauma informed. Others noted how staff in their services were changing the way they talked about trauma. Challenges to integrating trauma-informed care were also noted. One barrier was perceived inability to change certain standardized practices that, due to existing policies, were not subject to modification or were standards put in place by national boards. Some participants wondered about how to best integrate TI-FC care into such externally mandated practices.

#### 3.3.3 Engagement of Clients/Families and fathers/Coparents

A variety of strategies for engaging and involving caregiving adults in the continuum of care were reported, with changes necessarily specific and tailored to the practices of the different partner agencies. For example, Obstetrics and Gynecology Care began inviting fathers to a specific longer prenatal visit typically attended by mothers alone (if the mothers so wished), developing father-friendly flyers explaining prenatal procedures for mothers that were shared at that visit. Other positive new developments described included gathering of informal feedback and satisfaction surveys, involvement in treatment planning, and greater involvement of families at community events. Awareness of involving families in an advisory capacity was reported in one agency. Specifically, regarding enhancements to father and coparent engagement, participants at each agency noted making strides in being more conscientious about involving men and fathers -- and attributed this greater awareness to the initiative training.

#### 3.3.4 Community awareness, organizational readiness and vision

Participants expressed interest in learning from leadership more about the purpose and future goals of TI-FC care and developing initiatives. They expressed interest in learning how the Collaborative would take the training to the next level, both in terms of expansion and sustainability. Many asked (enthusiastically), “What’s next?” (e.g., practical knowledge application, sharing of information, resources, and strategies across agency partners). Perspectives regarding leadership involvement in promoting TI-FC care ranged from viewing “higher” leadership as minimally present or active in promoting the integration of TI-FC practice, to perceiving leadership as open and receptive to staff needs. One recommendation concerned the continuation of visibility among the agency’s executive leaders in advocating for and promoting TI-FC care. A shared theme across several groups concerned the importance of sustaining training efforts to avoid reverting “back to business as usual.” Participants also emphasized the need to broaden community partnerships and to expand education efforts within the agency and out in the community.

Focus group results were presented to agency partners to reflect on and make decisions about future internal action and change. Overall, based on focus group results, partners were encouraged to:

- Ensure all staff impacted by the initiative received regular communications describing initiative goals and accomplishments, such as newsletters/email communications, and that executive leadership were visible in these efforts.
- Improve trainings by delving into more nuanced topics such as the impact of racial and other forms of trauma specific to particular populations of clients and families.
- Add staff support at the trainings in the event re-traumatization occurred.
- Provide staff with organized assemblies of training materials and resources.
- Add more activities to support knowledge integration and practical application of learned material following training activities.
- Explore the feasibility of developing brief tools and practices that can supplement standardized procedures.
- Continue emphasizing the complementary nature of TI-FC care to existing practice.
- Explore opportunities for supporting a meaningful and authentic involvement of families in a formal, advisory capacity.
- Continue providing additional strategies for supporting father/coparent involvement.

## 4 Discussion

As evidence of the long-term impact of trauma and early adversity during children’s first 3 years of life has mushroomed, health care providers have increasingly sought to develop more grounded approaches to trauma-informed care. However, emerging evidence indicates that intentional and broad-based changes to organizational policy and culture are needed before health care settings can become truly trauma-informed and ready to responsibly address aftereffects of trauma among clients and staff (Menschner and Maul, 2016). Transformations toward becoming trauma-informed organizations that respect and include children’s fathers in their work need guidance and leadership from senior staff and management. Concurrently, the front-line workforce must also take part in transformative efforts to maximize buy-in throughout the organization. Involving multiple lead agencies in making such changes simultaneously and collaboratively can synergize changes within systems of care and maximize impact for fathers and their families in communities served.

The case study presented in this report found significant initial success in adapting approaches to care in serving children 0–3 and their families. We proactively engaged several lead agencies and organizations that maintained the most saturated touch in the lives of families from pregnancy through the child’s first 3 years. We also engaged all partners in purposeful planning in which universal aims, transformational principles, and common on-the-ground shifts were synchronized across an intensive two-year implementation period. This deliberative and collaborative multi-agency team approach enabled coordination of organizational assessment, staff training and consultation, self-monitoring of organizational shifts, and problem-solving of obstacles and solutions. The Collaborative’s particular success in serving fathers owed, in large part, to participating agencies all successfully completing comprehensive and multi-faceted analyzes of organizational culture -- then using products of this evaluation to make calculated and customized changes within their agency. Preliminary data presented in this report indicate that considered across agencies, important cultural shifts took hold in agencies and signified changes not just in father engagement, but across multiple key domains.

The infrastructure of the initiative helped agencies systematically approach assessment, self-review and reflection, staff training, and competency-building among senior staff, all enhanced by improvements in reflective practice. The commitment to regular participation in review meetings, sending the same staff and delegates across time, and coming prepared to discuss successes and hiccups held organizations accountable during the intensive change period. The camaraderie of multiple organizations investing similar efforts and producing customized innovations afforded unique and, in some ways, unparalleled opportunities for brainstorming, emulation, and experimentation. The ongoing exposure of staff and supervisors to how the initiative was taking hold across sister organizations during the training series events was also unusual and impactful. Personnel across multiple agencies gathering in the same rooms for core trainings, hearing how father engagement and other TI-FC issues were being prioritized -- and playing out -- across different healthcare and related settings elevated everyone’s awareness of the endemic nature of ingrained practices. It also highlighted the promise of striking upon new ways of viewing and collaborating with fathers and families informed by a trauma-informed family-centered lens.

The guiding inspiration for this effort was questioning and challenging the narrow lens behind the typical approach to trauma-informed training in agency settings serving children birth to 3. Almost invariably, that lens is dyadic (child and one parent), at best (McHale and Phares, 2015). While we advocated that infants’ fathers be noticed and valued, such advocacy was not itself new – father engagement has been discussed for nearly 30 years in major federal initiatives. What was innovative was providing not just a conceptual blueprint for understanding but also “in-the-trenches” role plays and conversations reviewing how providers can properly – and also ineptly – approach fathers. Understanding the psychology of men and fathers is essential when the aim is to include them substantively in care plans (McHale and Jenkins, 2023). During live trainings, multi-agency staff were asked to - and proved capable of - reflecting upon and articulating why they’d left fathers out of current cases they were seeing, when fathers actually could have been involved. Gains in provider recognition that true trauma-informed care for infants requires outreach to and engagement of the multiple adults, or coparents, responsible for the child’s care and upbringing were seen in the cross-agency data presented above. Staff ratings of increases in their understanding were greatest for Domain 3: Father and Coparent Engagement in TI Care (Table 2).

These gains and benefits noted, the work reported here only began to scratch the surface of true organizational and systemic change. There were certainly major successes. All agencies implemented or augmented universal trauma screenings. Several agencies also made substantive changes to their clinical approaches to father and coparental engagement. For example, a substance abuse agency altered their intake questionnaires to ask men seeking treatment if they were fathers, expanding service options if they were, and expanded treatment groups – and staff competencies in leading groups - from “mommy and me” to “my family and me”. However, other agencies – particularly medical settings, but others too - reported greater obstacles.

Common in medical contexts was upper management disinclination to pursue more inclusive approaches, often citing confidentiality, charting, and billing conventions and constraints. Challenges were also encountered in the ready development of a desired single central intake and referral port of entry (through a Healthy Start Coalition) for referrals among agency partners. Legal concerns were cited regarding confidentiality protections in patient consents. While workarounds were struck upon for certain obstacles, others were not as readily navigated. Still, each agency did make considered and meaningful changes within the purview of that allowable by their own oversight boards and funders. Cultural shifts were also seen in the development of new, more inclusive client materials, even within medical settings, such as the aforementioned father-friendly flyers explaining prenatal procedures for mothers.

We cannot close without commenting on the costs of such work, and the value of having had a sponsor to help defray some of the genuine expenses associated with a time- and labor-intensive initiative such as this. As alluded to earlier, the project was sponsored by an area Foundation. The investment of planning funds to help compensate agencies for the allocation of time by upper-level management and decision-makers to attend planning and calibration meetings regularly was crucial. So too was allocation of funds for agencies to designate time for staff training, rather than billable clinical activities. The wholesale participation of unit staff in ongoing training might potentially have negatively influenced the financial health especially of smaller organizations, so the financial offset was helpful in those cases.

Foundation staff themselves even made time to attend group partner meetings where progress was charted. Because of their deep, informed understanding of the transformations being accomplished through the TI-FC Collaborative, they were well-situated to consider a Family Study Center request for a new arm of the community effort. Reflective trainings had uncovered staff concerns and occasional discomfort working across racial and cultural lines with fathers and families from non-concordant demographic groups. As a result, the Foundation augmented the TI-FC transformative project with additional new funding allowing both original TI-FC partners and new area providers access to training and consultation on casework with families, with a focus on race-based trauma. The initiative also offered support for BIPOC practitioners in the region.

Moreover, the extensive contacts with agency over the course of the TI-FC Collaborative identified a second competency concern harbored by staff – that they had never had training working with multiple caregivers simultaneously. This self-identified knowledge and skill gap has become a focus in a second, follow-on initiative currently being piloted with some of the same original TI-FC collaborative agencies. That initiative, which included an intensive planning phase involving organizational leadership, emulating the approach taken it this report, is situated to provide intensive organizational training and in-services, and weekly group and *ad-hoc* case consultation, for delivery of agency-customized brief coparenting consultations to families already being served by front-line providers (McHale, 2023).

We believe that the modest but pivotally important humanitarian investments of knowledgeable funders open to supporting dedicated activities that helped contextualize and expand the scope of the community’s systems change efforts are crucial. Funder-supported university-community partnerships -- especially when they are deliberative and inclusive of the major community partners already serving infants, fathers, and families -- stand to expand the existing knowledge base about system change and supports for higher risk children, families, and communities. In this regard, we note that agency leadership in the community served had already been meeting, often several times annually in various forums absent of funding, for over 12 years to focus on infant-family mental health. Hence a stage had been set to organize quickly and effectively once a funding opportunity presented itself. This model is one that can be realized in any community at no cost, and authentic, altruistic collaborations in the best interests of young children and their families are desirable to collective impact Foundations and funders.

We believe future efforts will be most effective when attentive to fathers’ and families’ lived experiences and past encounters with healthcare systems, guided by community voices, and attuned to needs of agency staff for protected opportunities to reflect upon and receive support for the challenges and occasional secondary traumatization they sometimes face. Such efforts are on the upswing, and the chronicling of their successes and challenges is necessary to continue to help broaden the collective impact of trauma-informed, family-centered work. TI-FC systems of care promise to increase and maximize the impact of coordinated supports in responding authentically to early childhood adversity and the sensibilities of fathers and families to cultivate meaningful, long-term change.

## Data availability statement

The raw data supporting the conclusions of this article will be made available by the authors, without undue reservation.

## Ethics statement

Ethical approval was not required for the study involving humans in accordance with the local legislation and institutional requirements. Written informed consent to participate in this study was not required from the participants or the participants’ legal guardians/next of kin in accordance with the national legislation and the institutional requirements.

## Author contributions

JM: Conceptualization, Funding acquisition, Writing – original draft, Investigation. DB: Data curation, Formal analysis, Methodology, Writing – review & editing. LN: Conceptualization, Funding acquisition, Project administration, Writing – review & editing. AJ: Writing – review & editing, Formal analysis, Writing – review & editing. LB: Resources, Supervision, Writing – review & editing.

## Footnotes

^1^For the community and partners involved, the initiative was dubbed a Trauma-Informed Family-Centered Collaborative, as not all agencies, partners and staff were familiar with the terminology Infant-Family Mental Health. The approach taken, however, was guided by and wholly commensurate with the relevance of coparenting theory and practice within the infant mental health field (McHale, 2007; McHale and Phares, 2015), and broadly speaking the efforts inculcated through this collaborative directly and materially supported infant-family mental health.
